# AIoT in Agriculture: Safeguarding Crops from Pest and Disease Threats

**DOI:** 10.3390/s23249733

**Published:** 2023-12-10

**Authors:** Pedro Blanco-Carmona, Lucía Baeza-Moreno, Eduardo Hidalgo-Fort, Rubén Martín-Clemente, Ramón González-Carvajal, Fernando Muñoz-Chavero

**Affiliations:** 1 Department of Electronic Engineering, University of Seville, 41092 Seville, Spain; pblanco@us.es (P.B.-C.); lbaeza@us.es (L.B.-M.); carvajal@us.es (R.G.-C.); fmunoz@us.es (F.M.-C.); 2 Department of Signal Processing and Communications, University of Seville, 41092 Seville, Spain; ruben@us.es

**Keywords:** Internet of Things (IoT), Wireless Sensor Network (WSN), NB-IoT, smart agriculture, Artificial Intelligence (AI)

## Abstract

A significant proportion of the world’s agricultural production is lost to pests and diseases. To mitigate this problem, an AIoT system for the early detection of pest and disease risks in crops is proposed. It presents a system based on low-power and low-cost sensor nodes that collect environmental data and transmit it once a day to a server via a NB-IoT network. In addition, the sensor nodes use individual, retrainable and updatable machine learning algorithms to assess the risk level in the crop every 30 min. If a risk is detected, environmental data and the risk level are immediately sent. Additionally, the system enables two types of notification: email and flashing LED, providing online and offline risk notifications. As a result, the system was deployed in a real-world environment and the power consumption of the sensor nodes was characterized, validating their longevity and the correct functioning of the risk detection algorithms. This allows the farmer to know the status of their crop and to take early action to address these threats.

## 1. Introduction

Each year, up to 40% of the world’s agricultural productivity is lost to the ravages of pests and diseases [1]. A significant proportion of this staggering loss can be attributed to the lack of timely detection and identification of these threats. In the past, this problem was visually detected, by observing changes in the appearance of the crop. However, this is a tedious and time-consuming process when trying to assess the condition of each individual plant.

To date in human history, there have been four agricultural revolutions [2]. The earliest of these was known as traditional agriculture, where individuals solely relied on manual labor and animal power to tend to their crops. In the mid-20th century, the second was characterized by the introduction of powered machinery systems, allowing a decrease in manual labor and an increase in productivity, coupled with the use of fertilizers and pesticides. The late 20th century marked the introduction of automated agriculture and monitoring crops. Finally, the most recent revolution incorporates the fundamental principles of Industry 4.0, involving advances such as Artificial Intelligence, the Internet of Things and Big Data, introducing new applications: smart metering [3,4] and artificial intelligence to optimize crop productivity [5,6], effectively addressing issues related to pests and diseases without resorting to conventional pesticide use. Other notable developments include intelligent irrigation methods [7], monitoring systems to manage smarter farms [8], the monitoring of water consumption levels [9] and efforts to reduce both environmental impact and resource depletion [10,11].

With regard to pests and disease risk detection systems, several solutions have been proposed. These include computer vision techniques that analyze images for signs of pest infestation [12] or disease manifestation [13]. There has also been research into plant diseases using aerial images taken by unmanned aerial vehicles (UAVs) [14]. In addition, some systems have been developed to collect environmental data to aid both pest and disease detection [15]. Some approaches even combine different technologies to address this problem [16].

The Internet of Things (IoT) and Artificial Intelligence (AI) have proven to be invaluable tools in this respect. By applying these two technologies together, a remarkable fusion known as the Artificial Intelligence of Things (AIoT) has emerged, showing immense potential. Some of the most interesting features of this new paradigm include:

- The availability of new low-cost sensors, which make it cheaper to create wireless devices and allow for a multitude of sensors adapted to the desired needs.
- Wireless communications, such as NarrowBand-IoT (NB-IoT) [17], which allow high coverage in rural environments.
- Low-power microprocessor systems, which ensure the longevity of AIoT devices.
- The ability to analyze situations and make decisions.

The main objective of this paper focuses on the successful implementation of a low-power and low-cost AIoT pest and disease risk detection system. It should be noted that several existing systems rely on image analysis, although with inaccurate results when applied to large areas, while others use expensive and high-maintenance UAVs to achieve the desired results. In addition, some systems collect environmental data using AI algorithms to facilitate the development of energy-efficient and cost-effective solutions.

This paper starts with an overview of the entire system, explaining how it works. This is followed by an explanation of the most important components of the system, namely sensor nodes and the server. In addition, emphasis is placed on the development of the algorithm used for the detection of pest and disease risks, as well as on the notification system in the case of risk detection. Then, it provides a description of the experimental results obtained and comparisons with other similar systems, to finally summarize the conclusions obtained from this work.

## 2. Materials and Methods

### 2.1. System Overview

#### 2.1.1. System Architecture

**Figure 1:**
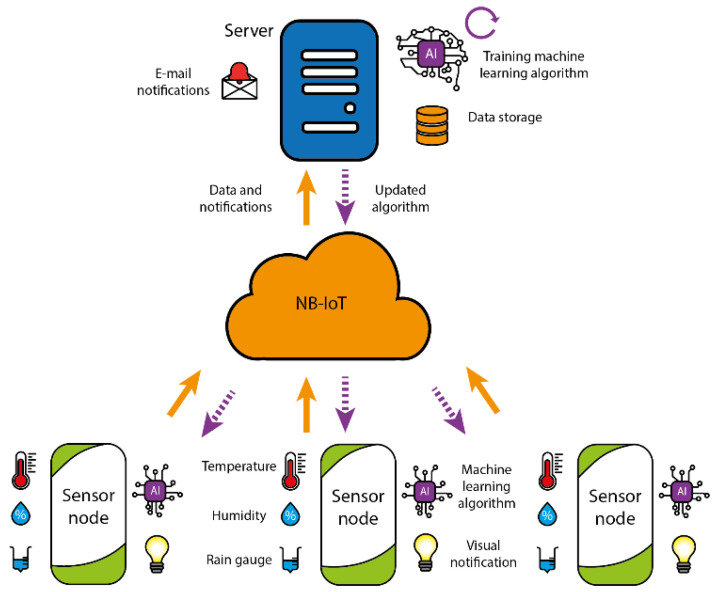
System architecture.

The system architecture is shown in Figure 1 where orange arrows represent uplink and purple ones downlink communications.

The system is composed of three blocks:

- Sensor nodes: The sensor nodes are tasked with collecting data from environmental sensors. Using the NB-IoT network, this information is then wirelessly transmitted to the server. In addition, these sensors are evaluated in real time using a machine learning algorithm. This is used to determine if any pest or disease threats are emerging in the agricultural landscape. In cases where risks are detected, notifications are immediately sent to the farmer, alerting them to potential problems in specific areas of their crop. A decision tree algorithm was chosen for this AIoT system because of its compatibility with low-power nodes and its innate simplicity for human comprehension.
- NB-IoT: The NB-IoT module acts as a wireless communication tool within the NB-IoT network, which is managed by mobile operators. This module establishes direct connections and is designed to provide sensor nodes with a long range and longer battery life due to its IoT-compatible infrastructure. This compatibility makes it an essential component for unattended devices deployed in agricultural environments, bringing significant benefits to these systems.
- Server: The server receives data from the sensor nodes and stores it in a database. This data is then used to continuously train the machine learning algorithm. It is important to note that the server also manages important risk alerts generated by the sensor nodes. These alerts are immediately sent to the farm owner, allowing them to react in a timely way and take the necessary measures to prevent any potential loss of crop or production yield.

#### 2.1.2. Description of System Functionality

**Figure 2:**
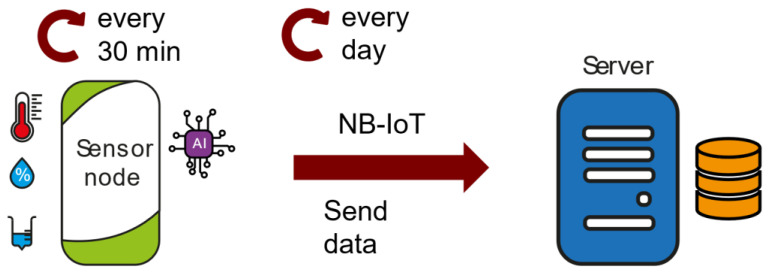
Data logger functionality.

**Figure 3:**
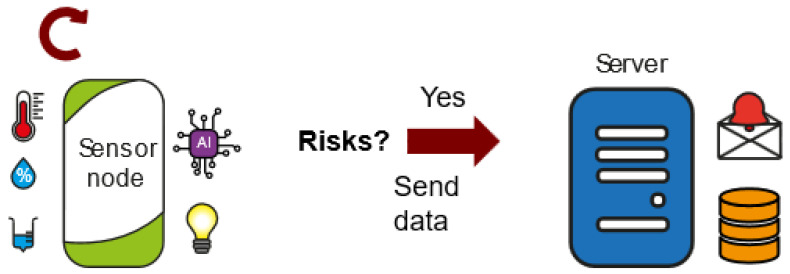
Risk evaluation and notification.

**Figure 4:**
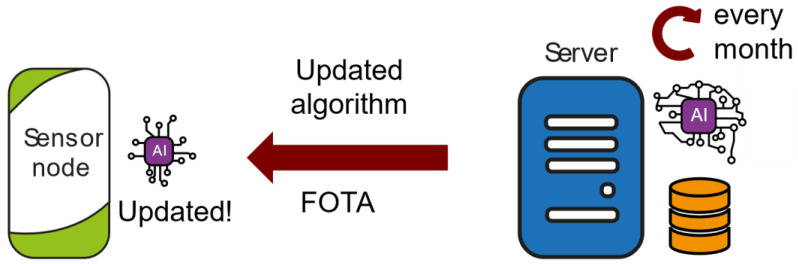
Training and updating machine learning algorithm functionality.

The sensor network has three different use functionalities:

- Data logger (Figure 2): Sensor nodes collect temperature, humidity, and rainfall data every 30 min. This information is stored in their memory. Every day, these data are sent to the server. Sending data every day was chosen as a compromise between the requirement for immediacy whilst minimizing power consumption, thus increasing battery life. A daily transmission frequency is adequate to monitor crops. If for some reason the sensor node detects a problematic situation, it would immediately send all the stored data information with an appropriate warning, as can be seen in the ‘risk assessment and notification’ functionality.

- Risk evaluation and notification (Figure 3): Each time environmental data are collected in the data logger functionality, the risk level of the area is evaluated. By default, there is no risk and the system acts as a data logger. However, if it detects a risk, it immediately sends a risk alert along with all the information from the sensors. In this case, the server receives the alert and manages the necessary notifications so that the farmer is aware that there is a problem on the farm. In addition, the device installed on the farm has visual warning lights that flash at different frequencies when they detect a certain level of risk in the area. This functionality enables a low-power and once-a-day solution that is also capable of detecting rapid changes and improving system responsiveness.

- Training and updating of the machine learning algorithm (Figure 4): Once a month, the server uses the collected environmental data to retrain each machine learning algorithm. These algorithms, which are specifically trained for each sensor node, are updated thanks to the Firmware Over The Air (FOTA) feature. This allows the algorithm to adapt to different terrain conditions, depending on where the device is installed.

### 2.2. Sensors Nodes

The sensor nodes were specifically developed for this work and they are made up of two different boards. On the one hand, the Core Board is responsible for the processing and control of the peripherals, and on the other hand, the Sensor Board carries out the sensorization. The core of the first board is a STM32L152RE microprocessor (ST microelectronics manufacturer), a microprocessor based on an ultra-low power ARM Cortex M3, with a clock of 32 MHz and able to operate between 1.65 and 3.6 V. This microcontroller allows us to run a real-time operating system (FreeRTOS), which helps to concurrently perform sensor data collection, machine learning algorithm execution and data transmission.

**Figure 5:**
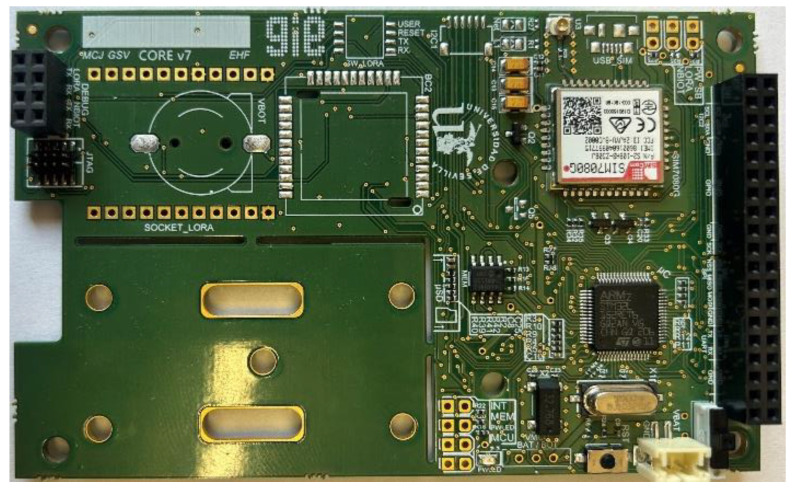
Core Board.

As additional peripherals to the microprocessor, the core board (Figure 5) is equipped with:

- EEPROM memory for storage of data acquired by the sensors.
- A SIM7080 NB-IoT transceiver (SIMCOM manufacturer), used to send the collected data to the server. It communicates with the microprocessor based on AT commands and a standby power consumption of 3 µA.
- SIM card, required for connection to the mobile NB-IoT operator network.
- Programming interface (JTAG).
- Access to the I2C, SPI and UART communication interfaces. These interfaces are essential for managing workflows.

ECONATUR agricultural expertise was used to identify which sensors are the most appropriate to identify pest and disease risks, obtaining the following sensors: temperature, humidity and crop precipitation. Market research for different sensors was carried out. These were chosen based on the following criteria: accuracy and energy consumption.

On the market, temperature and humidity sensors are usually included in a single sensor. Table 1 shows the different sensors evaluated according to the characteristics mentioned above.

As all sensors had very similar tolerances and the resolution of 14 to 16 bits was not significant for our application, it was decided to prioritize the power consumption, choosing the SHT20 sensor, as it has the lowest power consumption.

Regarding the rain gauge, they are usually associated with complete weather stations, but a low-cost, low-consumption solution was needed. Therefore, the WH-SP-RG rain gauge with pulsed output was chosen, which minimized the cost and guaranteed low energy consumption.

Thus, the sensor node was equipped with a temperature and humidity sensor SHT20 and a WH-SP-RG rain gauge.

**Figure 6:**
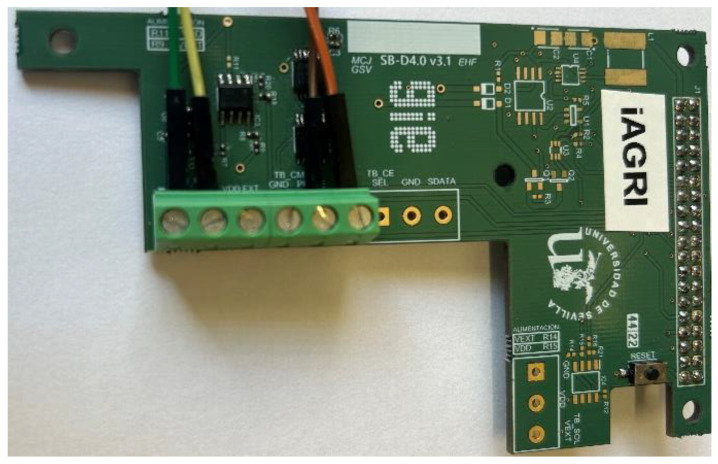
Sensor Board.

As can be seen in Figure 6, the sensor board designed has some non-welded components that are intended for additional tasks to be carried out during the overall project.

**Figure 7:**
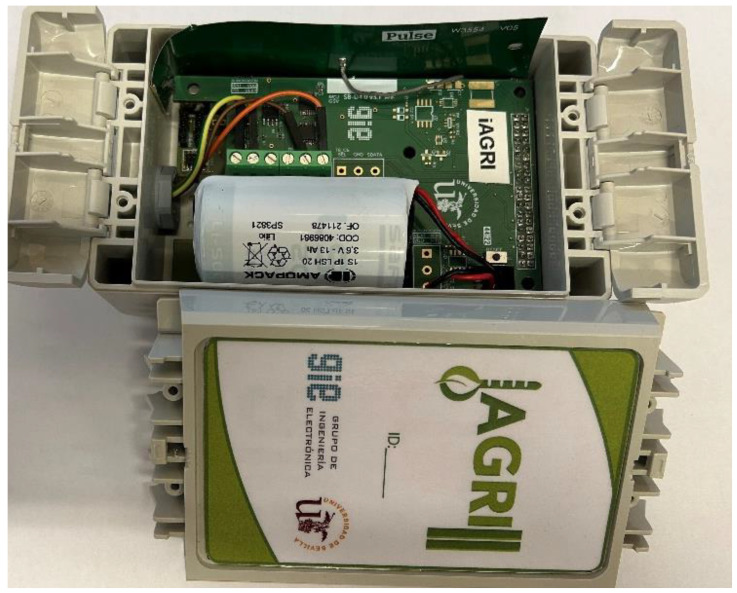
Sensor Node.

The sensor node, comprised of the two assembled boards, the battery (LSP33600-20F), the antenna and the sensors, is shown in Figure 7:

At the firmware level, drivers were created to manage each sensor and manage the external peripherals, as well as all the FreeRTOS tasks required.

**Figure 8:**
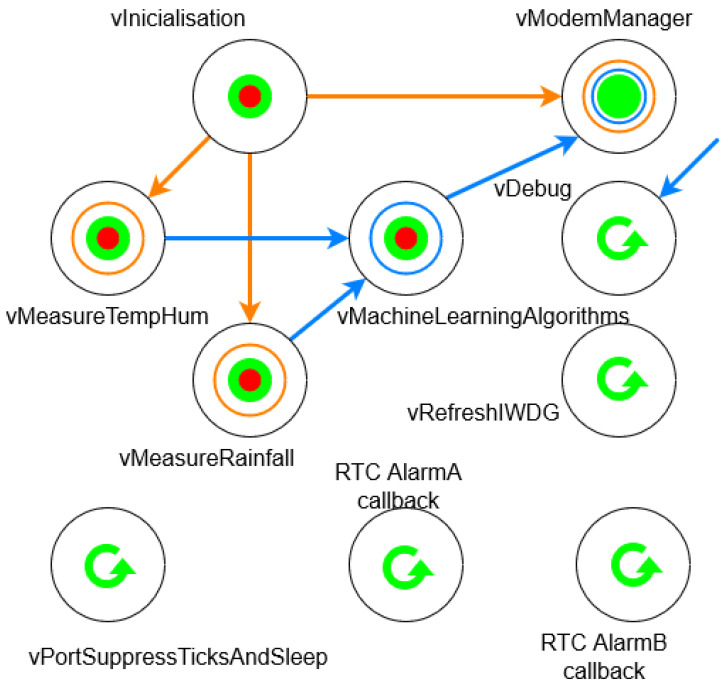
FreeRTOS tasks diagram.

The above task diagram (Figure 8), where blue arrows represent queues and orange ones, RTOS semaphores, can be described as follows: *vInitialisation* is responsible for initializing all the environmental sensors and the NB-IoT transceiver, as well as detecting whether FOTA is required. The *vMeasureTempHum* and *vMeasureRainfall* tasks then collect the temperature and humidity, and rainfall, respectively, and pass them to the *vMachineLearningAlgorithms* task, which is responsible for detecting whether there are pest and/or disease risks using the decision tree algorithms. If there is no risk, the system goes into standby mode, and if there is a risk, it sends it over NB-IoT via the *vModemManager*. In addition, data is periodically sent, whether there is a risk or not. Additionally, there is a debugging task (*vDebug*), watchdog management (*vResfreshIWDG*), low power management (*vPortSupressTicksAndSleep*) and management of different alarms (*RTCAlarmA-BCallback*). Thanks to this multi-tasking system, a high level of system reliability is achieved, enabling long-term deployment by allowing actions to be taken in the event of system failure, such as: *vResfreshIWDG task* restarts the system when the microprocessor goes into an unknown state, *vMeasureTempHum* and *vMeasureRainfall* tasks run sequences to reboot and/or shut down and power up sensors in the event of a lack of communication with them, and *vModemManager task* stores the data to be sent in the future in case the network is unavailable at that moment.

### 2.3. Decision Tree

A decision tree algorithm has the particularity that it is human readable and computationally simple, so it can be easily implemented in the sensor node. It is essential to be able to have an easy-to-integrate algorithm because the nodes will use edge computing to detect if there is any type of risk and quickly notify it.

**Figure 9:**
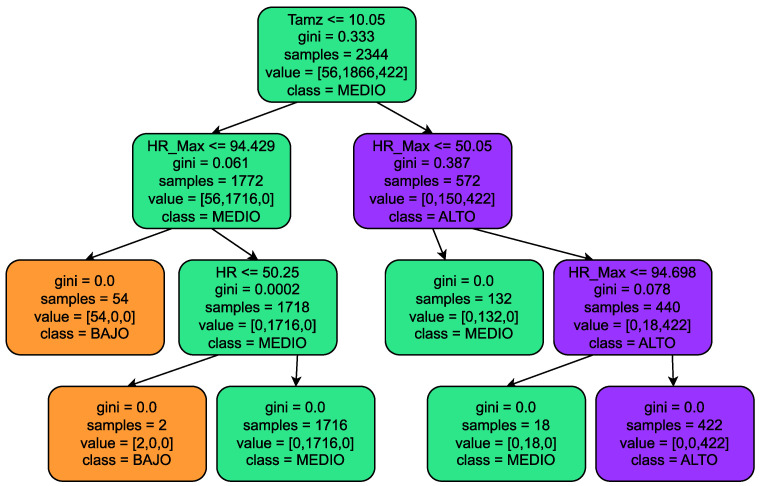
Decision tree for disease risk evaluation. Tamb = ambient temperature, HR = relative humidity and HR_Max = relative maximum humidity in the last 24 h. The algorithm outputs an assessment of risk, i.e., BAJO (low), MEDIO (medium) and ALTO (high).

The algorithm was initially trained using databases from meteorological stations, such as that of IFAPA [18], and using the knowledge of agricultural experts for labeling pest and disease risks. The input features of the algorithm are current temperature, maximum daytime temperature, current humidity, maximum daytime humidity and current rainfall. Finally, several decision trees were adjusted to detect a certain number of pests and diseases at different depths and sizes. Figure 9 shows a shallow decision tree to understand how it works through if other conditions with different environmental variables are involved. In general, the prediction of the algorithm is a certain pest/infestation risk level classified into three types: low, medium and high. An example is shown in Figure 9 of a decision tree evaluation with Tamb = 24, HR = 10, HR_Max = 40, assuming a medium level of risk for diseases.

Once the sensor was installed, the data collected were used by the server to further train the algorithm and improve it. In this way, it was possible to update the nodes with these remotely trained algorithms. The server is prepared to retrain the algorithms, which can be remotely updated on the sensor nodes at any time. The re-trainability of the algorithms gives the advantage of making new algorithms that can be adapted to the emergence of new pests and/or diseases based on the labels established by the agricultural experts.

The threshold of the decision trees will change over time. Thus, each sensor node will have its own specific decision tree. Depending on the distribution of the sensor nodes, they will be able to obtain different temperature, humidity and rainfall values, especially in large crops, so the pest and disease risk assessment will change. This feature allows the algorithm to adapt to the type of terrain, type of crop, type of pest, type of disease, climate, etc.

The sensor node, through the algorithm, sends notifications as soon as a change in risk level is detected, as opposed to waiting an entire day to send the information to the server.

### 2.4. Server

**Figure 10:**
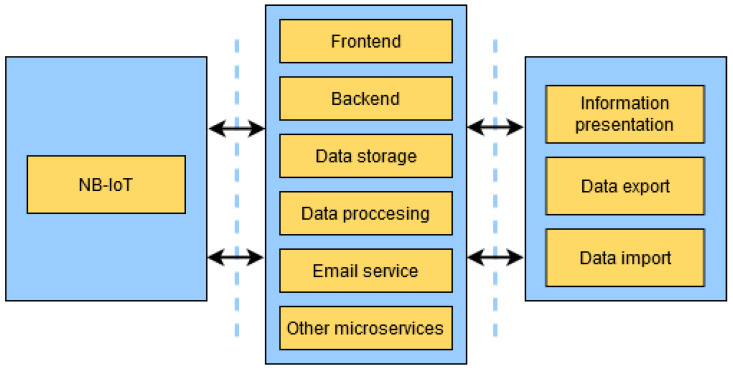
Server functional block diagram.

With regard to storage, data exploration, analysis and visualization of the information received by the remote nodes, a microservices architecture is available to guarantee scalability and isolation between the different applications involved in the process. Figure 10 shows a diagram with the basic microservices architecture involved in the information management system, as well as other intermediate middleware resources useful for the correct development of the system (security layers, load balancing management, reverse proxy, etc.).

The first block belongs to the sensor nodes deployed in the crop, which send sensor data via the NB-IoT network to the server. The data are sent every day unless a certain type of risk has been detected.

The server module, which forms the core of this section, is composed of microservices (applications) that operate in isolation but are able to communicate with each other. The main services deployed in the server are:

- Data storage: It oversees the storage of all data collected by the sensor nodes, both for visualization and to train the decision tree algorithm.
- Data processing: This microservice performs the whole algorithm retraining procedure, which is executed every month and generates new algorithms to be updated, through FOTA, in sensor nodes.
- Email service: It is responsible for building and sending weekly e-mail notifications on the status of the environmental variables sensed, and for sending notifications of pest and/or disease risk detection.
- Frontend: The frontend microservice is responsible for providing the necessary web interface so that the user can easily access the contents stored on the server.
- Backend: It supports the frontend operations, making any data it requires available and ensuring that all requests are secure.
- Other microservices: This paper is focused on the main features of the system, however, other microservices are executed in parallel, such as security layers and load balancing management.

Finally, there is a block for the presentation of information to the user and support for the export of original and system-processed data, as well as importing external data.

**Figure 11:**
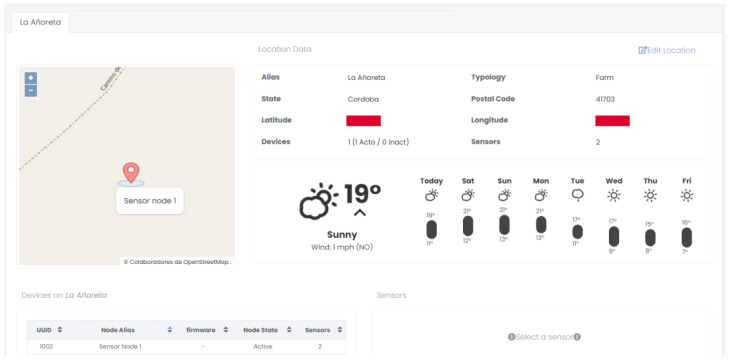
Developed graphical user interface.

**Figure 12:**
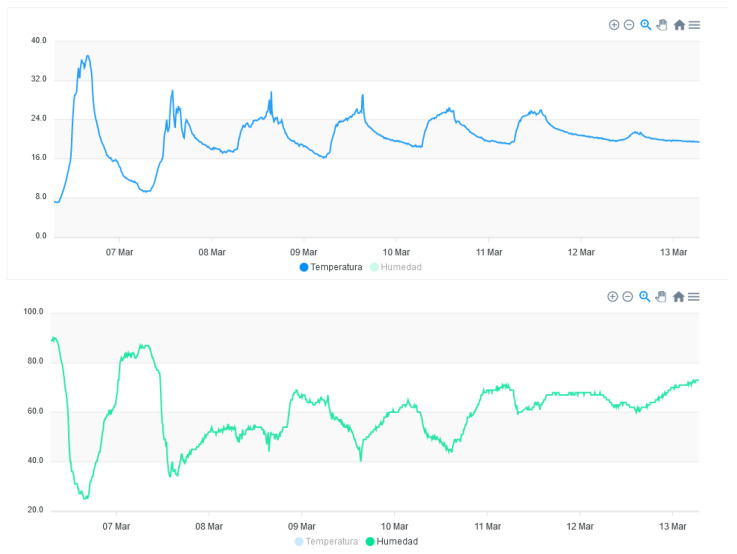
Temperature and humidity measurements obtained on the platform.

The web interface is presented in Figure 11. It shows the geographical area where some of the terminal nodes created have been located. Other features integrated in the website are visualization (Figure 12) and the export/import of the data.

On the web page, the data acquired from the different sensors are displayed. For instance, in Figure 12, the graphs of the temperature data in degrees Celsius (upper chart) and humidity in percentage (lower chart) over a period of one week are shown.

The ability to export the data was introduced to allow them to be displayed on other media and/or to create new machine learning algorithms that can be used in the sensor nodes, as well as other functionalities.

### 2.5. Notification System

A key issue for this system is to warn the farmer, as early as possible, when there is a pest and/or disease risk. The notification system must be adapted to the farmer’s needs and, at the same time, be quick, easy and intuitive to use. In this case, two different types of notifications have been developed:

- E-mail: Notifies, once a week, the pest and disease risks that have occurred during that full week, providing the overall risk of each day and graphs with the profile of the risks obtained throughout each day. It also includes information on the evolution of the monitored variables.

**Figure 13:**
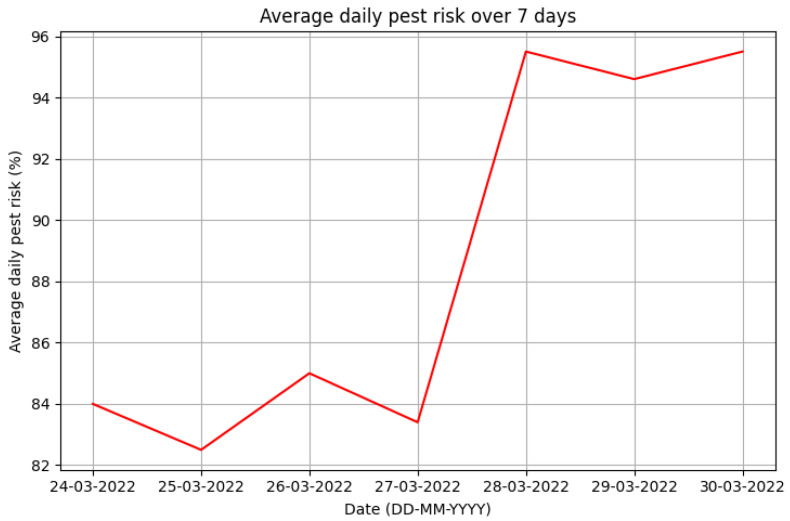
Graph of pest risk levels over 7 days (extract from notification e-mail).

An extract of the email notifications (Figure 13) is presented in which the percentage of the average daily pest risk for seven days is shown.

- Visual notifications: As farmers may not have internet access, an offline solution is offered using visual notifications. A red LED blinks, with an active time of 200 ms, at a frequency of 30 s when medium risk is detected and at 15 s when high risk.

Both types of notifications coexist, leaving the farmer with two different ways to determine that everything is correct in their crop. Moreover, thanks to the storage of all notifications on the server, any type of alert (sound warning, Telegram bot, SMS, etc.) can be applied according to the needs of each farmer.

### 2.6. Power Consumption Analysis

To test the performance of the system, several field experiments were carried out.

The laboratory tests were focused on two key points: the correct data collection of all sensors and the energy consumption of the terminal node.

A calibration of the temperature and humidity sensor was performed using a commercial temperature and humidity sensor, RS-1367. Once this was completed, it was verified that the readings of the sensors were correct. At the end of the calibration, it was possible to carry out data transmission tests to the server, in order to ensure the correct operation of daily transmissions. It was verified that the daily transmissions were fulfilled with all sensor samples correctly collected, with their corresponding time stamps.

**Figure 14:**
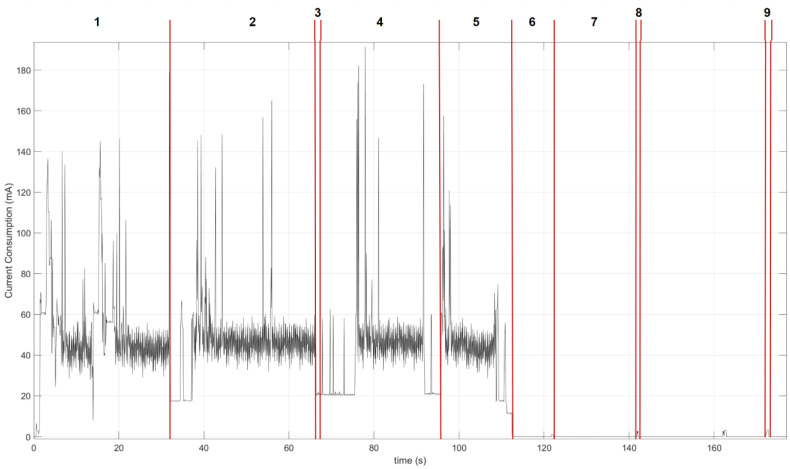
Node power consumption analysis.

Secondly, the power consumption of the device was verified with Keithley 2450 SourceMeter (Keithley manufacturer), shown in Figure 14. This analysis was divided into eight different phases, separated by red lines, as follows:

1. The first phase is responsible for verifying the connection to the destination server. To do this, the connection to the service operator is made through its APN and the connection is checked for security by means of SSL certificates. This takes approximately 32 s with an average consumption of 52 mA.
2. As can be seen in Figure 14, this is the longest phase of the node’s duty cycle. It has a duration of 34 s during which the connection to the MQTT broker (secured by a username and password) used for the transmission of data through a TCP connection that guarantees the delivery of the information is carried out. This phase is also used to check whether there is new firmware to update the node by means of the FOTA (Firmware-Over-The-Air) functionality available. This phase is characterized by an average consumption of 45 mA.
3. In contrast to phase 2 and due to the capacities of the microcontroller, this phase is the shortest of the whole active cycle and is responsible for taking measurements, collecting the data stored in local memory and local processing. It uses an average current of 20 mA for 0.67 s. This phase will be repeated every 30 min, with a 2 mA for 0.67 s, with a marginal consumption cost for the overall calculation of consumption.
4. Once the information has been collected, the node creates the subscription to corresponding MQTT topic to send the data. Once the data have been sent, the unsubscribe action of the topic is carried out. This phase is carried out in a period of 29 s with an average consumption of 38 mA.
5. Phase 5 is responsible for disabling all possible processes and the configuration of the microprocessor and its peripherals, as well as the NBIoT transceiver in ultra-low power mode. This process requires a time of about 17 s and a consumption of 42 mA on average.
6. During this phase, the device is in the low-power mode induced in phase 5. This phase will be interrupted by phase 7 every 20 s, at which time the WDT of the microprocessor is powered up. It is important to note that the node will be active during most of this phase, hence the importance of minimizing the power consumption of this phase as much as possible. In the present development, it has been reduced to 5 µA.
7. This is the power supply phase of the WDT, which periodically interrupts phase 6 to ensure that the device can recover from unknown or error conditions. This action is carried out for a time of 0.42 s and involves an average consumption of 1.21 mA.
8. The detection and quantification of rainfall by the node does not respond to any periodic or predictable pattern: it is totally conditioned by weather conditions. At this point, the device has a hardware interrupt that is activated by the sensor mentioned in Section III during which an increase of 3 L/m^2^ of rainfall is added to the local history (to be transmitted in the next transmission to the server). This action implies an average consumption of 2.15 mA during 0.6 s.

Once the power consumption of each phase is known, the autonomy of the node depends on two main factors: the frequency at which data are transmitted and the capacity of the selected battery, in our case 17,000 mAh.

On the other hand, the number of times phase 8 is activated directly depends on the amount of rainfall at the deployment site. According to [19], Colombia is the country with the highest rainfall in the world, with an amount of 3240 L/m^2^, while in Spain it is 535 L/m^2^ [20]. This would mean a consumption of 1.28 mAh (in 1 year) in the worst case and 0.21 mAh per year in Spain, which would have the effect shown in Table 2 on the autonomy of the node.

Although one daily dispatch could be considered sufficient, given the nature of the information being measured, Table 2 respectively shows the autonomy of the nodes in case of two or four daily dispatches of information. If we consider 5 years of autonomy necessary in order to amortize the maintenance costs, we can see how this condition is met in every case, even in the rainiest area of the world with a daily dispatch. Therefore, the node design can be considered validated in terms of energy consumption.

Once all the tests had been verified, the device was to be installed, after carrying out coverage tests in the location to be installed, with different mobile operators. The study was carried out with two different operators. These were both determined to be suitable and, consequently, the farm was considered suitable for this type of technology.

### 2.7. Deployment in a Real-World Environment

**Figure 15:**
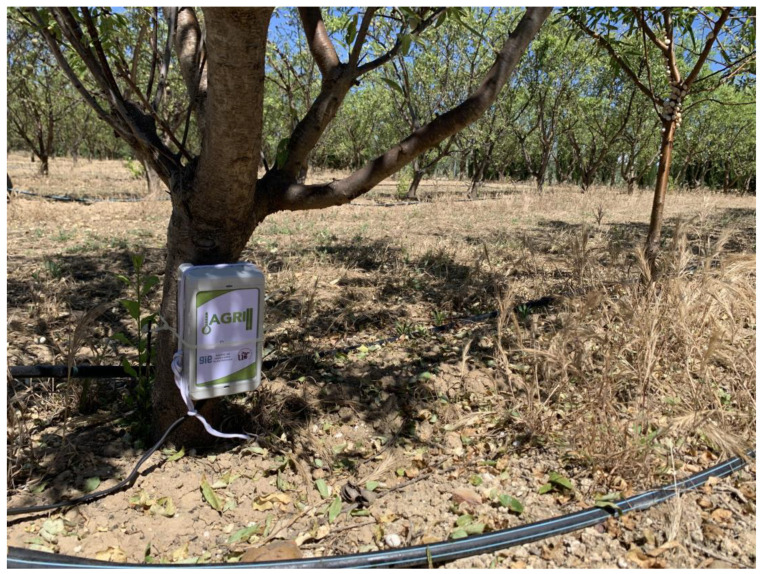
Sensor node installation in almond tree crops.

**Figure 16:**
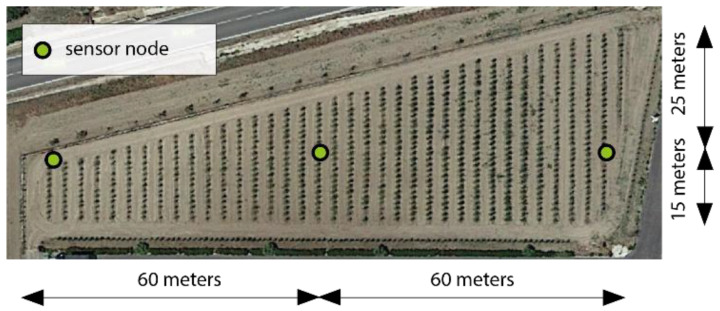
Distribution of sensor node network on the crop.

Once it had been tested in the laboratory, the system was deployed in a real-world environment, on the experimental farm la Añoreta, located in Santaella (Cordoba, Spain) with a surface area of 6 hectares. This farm is owned by the agricultural company ECONATUR. The sensor nodes were deployed on the farm (Figure 15) according to the distribution shown in Figure 16.

## 3. Results

Following the deployment of the sensor nodes in la Añoreta (Cordoba, Spain), tests were carried out with the aim of validating all system functionalities: data logger; risk assessment and notification; and the training and updating of the machine learning algorithm. In addition, the installation in a real environment was used to test the robustness of the system: the durability of the equipment, the battery life, correct data transmission in areas with a tendency for low mobile coverage and management of the server database. The tests were carried out between February and May 2022, in an almond crop.

In the months prior to the installation of the system, it was decided to collect data from the nearest IFAPA weather station (Santaella) to the crop where the sensor network was to be installed in 2022 in order to train the decision tree algorithm. When sensor nodes were deployed, algorithms were run and results were obtained in which there was a high pest risk between April and May. These results were presented to the experts, who reported that aphids were recorded on the crop during that period.

In February, the risk of the crop was evaluated, remaining healthy during the dermalogical stage of flowering and fruit set, with no incidence of any type of disease or pest. During this time, it was verified that the sensor nodes identified the risk as low in both cases (pest and disease). In addition, it was verified that data transmissions during that month were correct, with no loss of data. There were no false positives and none of the sensor nodes had to be reprogrammed.

**Figure 17:**
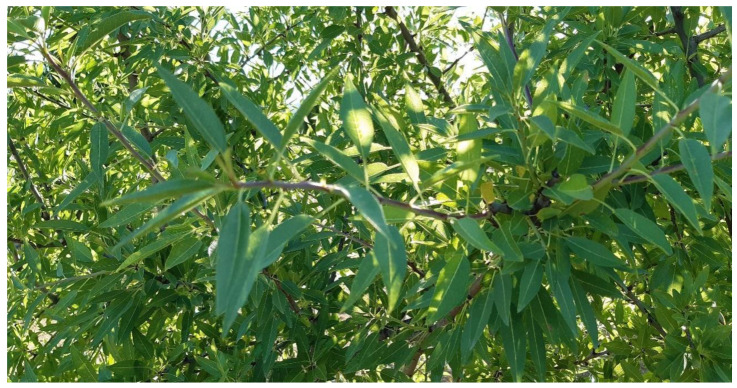
Almond trees without any pests and diseases.

Figure 17 shows that there were no signs of pests and diseases anywhere, with the leaves of the almond trees showing a uniform green colour.

This trend continued until 20 March 2022, when the sensor nodes started to report medium pest levels. Upon detecting this risk, the farmer went to check the farm and agreed that this coincided with the start of positive adult aphid counts, although at very low levels (one to three aphids per leaf).

**Figure 18:**
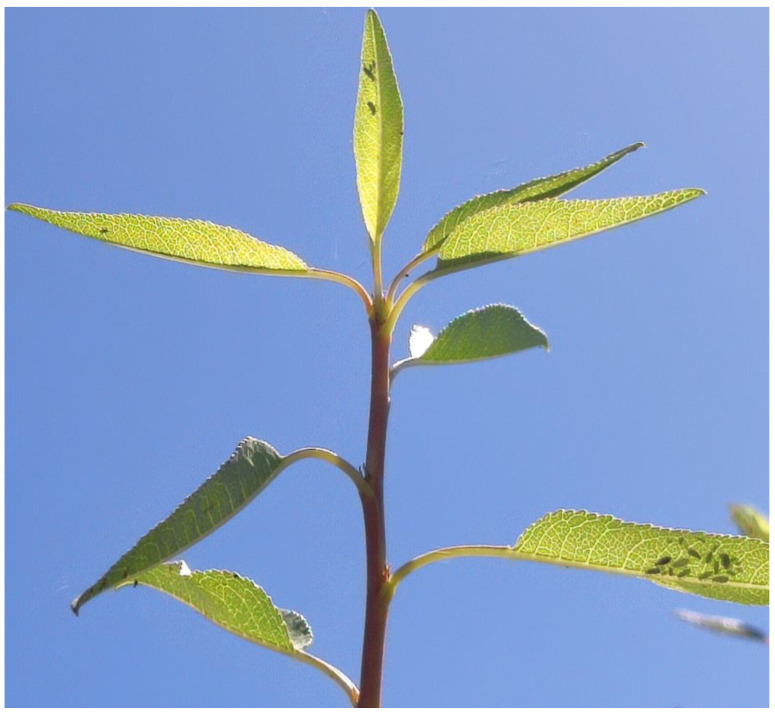
Almond tree with a few aphids on some of its leaves.

These aphid levels can be seen on the top leaf of Figure 18, which contains three aphids and a small colony of thirteen green aphids detected on the lower right leaf. 

On the days following the medium risk alert, the sensor nodes continued to maintain the medium risk level. This trend continued until 30 March, when it changed to a high pest level. On the following days, the farm was visited to verify the presence of aphids in the almond trees.

**Figure 19:**
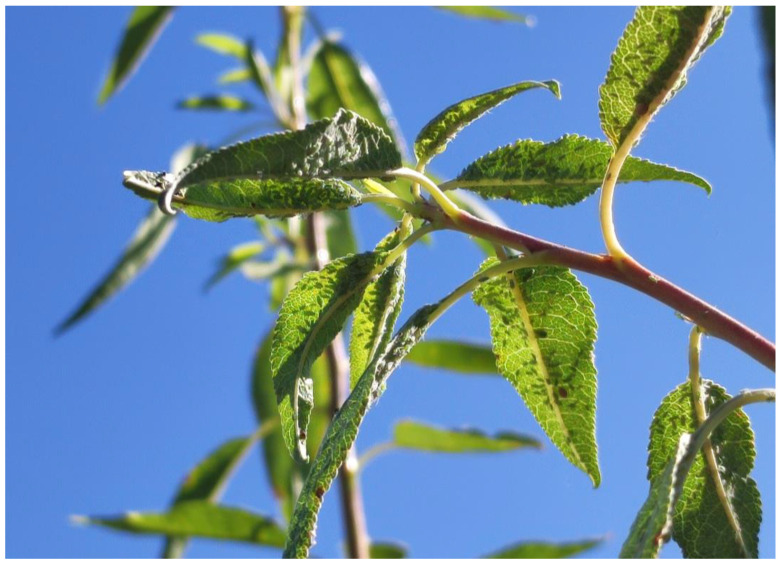
Almond tree completely infested with aphids.

In Figure 19, leaves are infested with aphids, visualising colonies of more than 40 per leaf. In this way, it was possible to confirm that the sensor nodes were correctly warning of the possible risks that could appear in the crops. 

During these months, the algorithm was remotely updated to check the correct performance of this functionality, with satisfactory results. Furthermore, it was verified that all the devices correctly worked, without the need to replace batteries, no problems were detected in coverage failure, and no samples were lost.

## 4. Comparative Study and Discussion

In this article, a comparative study of the developed system with previously proposed systems was carried out. Table 3 compares the main characteristics of the AIoT systems.

Comparing these systems, the use of edge computing in [22] and this work stands out, as the system does not depend on the server for the execution of the algorithm. Additionally, the system by Chen et al. lacks disease detection. While N. Materne et al. [23] used ZigBee, the three other systems use LoRa, and this work employs NB-IoT. NB-IoT, in contrast to ZigBee and LoRa, has the advantage that it is a self-managed network, being operated by the mobile operators. In this case, this network is available in almost 100% of the Spanish territory, with very competitive prices.

The algorithms presented in this work are retrainable, so they have high flexibility to changes such as new pests, new diseases, the type of crop, the type of terrain, etc. If the algorithm detects an increased risk, the system alerts the farmer. An important point is the presence of visual alerts in the system presented in this article, which is essential for the farmer not to be completely dependent on the Internet. It is understood that different farmers will want different methods of notification. As all alarms are stored on the server, the developed system allows the integration of any type of alarm, either physical (e.g., audible alarm), information technology (e.g., Telegram bot) or mobile (e.g., SMS).

This work permits the use of edge computing techniques, minimizing the power consumption of the sensor node, and does reduce the response time when notifying of a certain risk in the crop. The evaluation time is 30 min, close to the other systems. On the other hand, the transmission interval increases by up to one transmission per day. In addition, the number of sensors has been minimized, only using those that experts have considered necessary for this application. This results in a competitively priced, low-power and low-cost device.

## 5. Conclusions

This work demonstrates that it is possible to develop a pest and disease risk detection system using AIoT technology by developing low-power and low-cost sensor nodes. To this aim, all system functionalities (data logger, risk assessment and notification, training and updating of the machine learning algorithm) were validated with satisfactory results in a real-world environment. 

In addition, a decision tree algorithm is proposed capable of detecting the pest and disease risk levels in crops. ECONATUR agricultural experts have determined that it is possible to assess pest and disease risks based on crop temperature, humidity and rainfall measurements, minimizing the number of sensors required and therefore reducing the cost of the sensor node.

Thanks to continuous data acquisition, this system can continuously improve the algorithm and be remotely updated. Having a sensor nodes network in the crop allows for the detection of the location where the risk has emerged and will make it possible to study the evolution of pests and diseases in the crop. The advantage of retraining and updating algorithms allows for an adaptation to new pests and diseases, as well as different types of terrain and/or crops.

This system employs edge computing techniques, increasing transmission intervals via NB-IoT and uses a minimized sensor suit, allowing for a low-power, low-cost solution. The system also provides a high level of responsiveness to changes that may be detrimental to the crop, as under risk-free conditions the transmission rate may be one day, but if a risk is detected, the transmission is immediate. The risks detected are communicated to the farmer by means of alerts. Two are proposed in this work: visual alerts and e-mail alerts. However, thanks to the reception of risk reports, the server is ready to implement any other type of alert that a farmer may request.

With the results obtained, the feasibility of AIoT systems used to detect pests and diseases in crops was demonstrated, providing early risk warnings of crop issues. In this way, it allows the farmer to quickly act and minimize annual production losses.

## Figures and Tables

**Table 1:** Evaluation of temperature and humidity sensors.

Sensor	Resolution (bits)	Temp. Tolerance (°C)	Hum. Tolerance (%)	Power Consumption (µA)	Price (€)
SHTC3	16	±0.2 °C	±2%	Active: 430 Sleep: 0.6	1.05
HDC1080DMBR	14	±0.2 °C	±2%	Active: 710 Sleep: 0.1	3.84
SHT30-DIS	16	±0.2 °C	±2%	Active: 600 Sleep: 0.2	2.97
SHT20	14	±0.2 °C	±3%	Active: 300 Sleep: 0.15	4.57

**Table 2:** Autonomy based on the number of daily data transmissions.

Daly Data Transmissions	Device Autonomy (Years)	Autonomy in Colombia (Years)
1	22.14	22.1
2	13.31	13.28
4	7.40	7.38

**Table 3:** Comparative with others AIoT Systems.

Features	Ching-Ju Chen et al. [21]	Olivier Debauche et al. [22]	N. Materne et al. [23]	T. T. Win et al. [24]	This Work
Sensors	Temperature, humidity, mobile phone, UAV	Temperature, humidity, barometer, rain, gauge, …	Air temperature, air humidity, CO_2_, illumination intensity, soil temperature, soil humidity, soil moisture, leaf wetness	Temperature, humidity, pressure, sunlight level, water level	Temperature, humidity, rain gauge
AI algorithm execution	Server	Node	Server	Server	Node
Detect pests	Yes	Yes	Yes	No	Yes
Detect diseases	No	Yes	Yes	Yes (with images)	Yes
Alerts via internet	Yes	Yes	No	No	Yes
Visual alerts	No	No	No	No	Yes
Evaluation period	Each 1 h	Each 5 min	Each 30 min	-	Each 30 min
Transmission interval	Each 1 h	Each 5 min	Each 30 min	-	Each day
Network	LoRa	LoRa	ZigBee	LoRa	NB-IoT
Autonomy	-	-	-	-	17 years

## Data Availability

Data are contained within the article.
